# Fire Is Associated With Positive Shifts in Bumble Bee ( *Bombus vosnesenskii* ) Body Size and Bee Abundance in the Southern Sierra Nevada Mountains

**DOI:** 10.1002/ece3.70821

**Published:** 2025-04-07

**Authors:** Claudinéia P. Costa, Natalie Fischer, Melissa Arellano, Claudette C. Torres, S. Hollis Woodard

**Affiliations:** ^1^ Department of Entomology University of California, Riverside Riverside California USA

**Keywords:** body size, *Bombus vosnesenskii*, bumble bees, fire, meadows, Sierra Nevada Mountains

## Abstract

Climate change is increasing the frequency and severity of wildfires worldwide. Although wildfires are typically viewed as destructive, emerging research suggests they may have benefits for some species, including some pollinators. One reason for this is that wildfires can increase floral resource availability in the years immediately following the burn, potentially creating more favorable conditions for pollinator foraging and reproduction. In this study, we focused on how the 2021 KNP Complex Fire impacted the bumble bee *Bombus vosnesenskii* in the Southern Sierra Mountains, where the effects of fire on this pollinator species have not been previously explored. Consistent with bumble bee studies in other areas, we found an increase in the size of *B. vosnesenskii* workers in recently burned areas. This effect was detectable despite a limited number of sampling events and locations in our study, and irrespective of the habitat type (meadow vs. forest) in which sampling occurred. We failed to detect increased floral resource availability (abundance or diversity) in burned areas but did observe unique floral communities in burned areas. Our findings contribute to our growing understanding of fire's impact on pollinators and support the broad idea that fire might have benefits for some organisms.

## Graphical abstract

Wildfires increase the abundance and size of bumble bees in the Southern Sierra Mountains. Wildfires may benefit bumble bees by enhancing floral resource availability and increasing their populations.

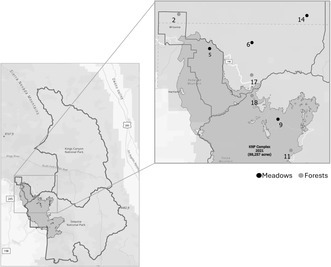

## 1 Introduction

Throughout human history, wildfires have been recognized as a fundamental, cyclical form of natural disturbance in many ecosystems (Bond and Keeley 2005; Lake et al. 2017). Climate change‐driven warming and drying of many parts of the world, along with fire suppression practices, are leading to dramatic shifts in global wildfire dynamics. Fire frequency, severity, and extent have all increased, particularly in the last few decades. This pattern is expected to intensify (Westerling et al. 2006; Miller et al. 2009; Flannigan et al. 2013; Jolly et al. 2015) and cause broad‐scale negative impacts across ecosystems. These impacts include habitat destruction, loss of biodiversity for some taxonomic groups, and disruption of critical ecosystem services such as carbon sequestration and water regulation (Malhi et al. 2020).

Wildfires also, however, play critical ecological roles that can be interpreted as being beneficial for organisms and the ecosystems they inhabit (Pyne, Andrews, and Laven 1996; DeBano, Neary, and Ffolliott 1998; Bowman et al. 2009; Hoffman et al. 2021). These include the promotion of biodiversity by facilitating habitat diversification (He, Lamont, and Pausas 2019). For example, fires can clear dense vegetation, which enables sunlight to reach forest floors, stimulates seed germination, and supports the growth of a wider variety of plant species (Lamont and Downes 2011). Fires contribute to nutrient cycling by releasing elements like nitrogen and phosphorus, enriching the soil and supporting new plant growth (reviewed by Certini 2005). Fires can also create habitats for specialized species adapted to post‐burn landscapes, such as woodpeckers, which use dead trees for nesting and food, as well as certain beetles and rodents (Saint‐Germain, Drapeau, and Buddle 2004; Zwolak, Foresman, and Sullivan 2010). Additionally, in grasslands, savannas, and certain forest types, periodic fires maintain open landscapes by preventing the encroachment of woody plants and supporting species that rely on these environments, including grazers and ground‐nesting birds. Through these processes, wildfires play a vital role in sustaining natural ecosystems' diversity, health, and resilience.

There is also evidence that the habitat changes brought on by wildfires can have benefits for insect pollinators, which provide important pollination services in many of the earth's ecosystems (Ponisio et al. 2016; Kim and Holt 2012; Fontaine and Dajoz 2021). For example, in bees (Anthophila), higher abundances of both solitary (Nakas et al. 2023) and social (Mola et al. 2020) species can be found in recently burned areas, and at the community‐level, fire can also influence bee community composition (Ponisio et al. 2016; Carbone et al. 2019; Galbraith et al. 2019), for example, by increasing the number of generalists versus specialist species (Lazarina et al. 2016; Nakas et al. 2023; Peralta et al. 2017). Further, larger‐bodied bees of some species have been documented in burned areas (Lazarina et al. 2016; Mola et al. 2020; but see Nakas et al. 2023). These patterns suggest that burned areas change in how they support bees or attract them from surrounding regions. This may result from increased floral resource availability (Ponisio et al. 2016; Fontaine and Dajoz 2021) and nesting site opportunities following a fire (Lazarina et al. 2016; Mola et al. 2020). Indeed, there is some evidence that recently burned areas have higher levels of floral resource availability, although the evidence for this is mixed (Kim and Holt 2012), possibly because there are fire intensity thresholds beyond which fire has negative impacts on resource availability. Despite growing evidence that wildfires influence bee populations and communities, including even in some putatively beneficial ways, the impacts of fire can only be better‐predicted with more comprehensive knowledge of how fire impacts this ecologically important group (Roy and Sparks 2000; Potts et al. 2010; Bartomeus et al. 2011; Goulson et al. 2015).

The bumble bees (genus *Bombus*, family Apidae) are a group of largely generalist pollinators that encompass a wide geographic range across Holarctic regions and beyond, particularly more temperate or montane ecosystems (Williams 1998; Goulson 2010). There is existing evidence that bumble bees, including species like *Bombus vosnesenskii* , benefit from fire‐disturbed landscapes through enhanced availability of floral resources, increased nesting habitat, and reduced competition (Hatfield and LeBuhn 2007; Cane and Neff 2011). Fire can clear dense vegetation, promoting the growth of wildflowers and other plants that provide essential forage for bumble bees, supporting their diet of nectar and pollen (Brown et al. 2017). Furthermore, the open spaces created by fire improve nesting opportunities, as bumble bees frequently nest in abandoned rodent burrows and other ground cavities exposed by the reduction of undergrowth (Harmon‐Threatt et al. 2020; Galbraith et al. 2019). This body of evidence suggests that periodic fire disturbances can play a beneficial role in supporting bumble bee populations, promoting resilience and biodiversity in ecosystems adapted to fire cycles.

Wildfires are dramatically reshaping California's montane areas (Keeley and Syphard 2021). Since the 1950s, wildfires have rapidly increased in frequency and area in California, with most of the largest fires in state history occurring in the last few years. This includes the Sierra Nevada Mountains (Halofsky 2021), where climate change, large‐scale tree mortality, and other factors have precipitated the occurrence of larger wildfires, expanding areas subjected to burning (Goss et al. 2020; Gutierrez et al. 2021; Williams et al. 2023). We collected a snapshot of data to examine how the 2021 KNP Complex Fire influenced bumble bees in the Southern Sierra Nevada Mountains. The 2021 KNP Complex Fire is one of the most extensive fires in the history of the Southern Sierra Nevada Mountains and, in total, burned more than 88,000 acres. Bumble bees are important pollinators of many native plant species in the Sierra Nevada Mountains (Loffland et al. 2017; Cole et al. 2020), including in areas burned by the KNP Complex Fire. The Sierra Nevada region supports generally high numbers of bumble bees (Thorp, Horning, and Dunning 1983; Loffland et al. 2017), as well as high levels of species diversity, including rare species (Thorp, Horning, and Dunning 1983; Loffland et al. 2017; Fisher et al. 2022), relative to many other regions in California. We focused on exploring the hypothesis that fire can benefit bumble bees in this system, by assessing whether *B. vosnesenskii* are larger in areas burned by the KNP Complex Fire, relative to unburned areas. Larger‐bodied bees may reflect the translation of greater food availability into colony‐level production of larger workers. We also surveyed bumble bees (all species, indiscriminately) and honey bees ( *Apis mellifera* ) to ask whether burned areas have greater numbers of these bees. A higher abundance of bees following fire may indicate that the habitat has become more supportive, for example, by providing more food resources in the area to support larger colony sizes. Ours is the first study to examine fire's impacts on bumble bees in the Southern Sierra Nevada Mountains as a point of comparison to previous studies that have found positive effects of fire on bumble bee abundance (Tarbill, White, and Sollmann 2023), size (Mola et al. 2020), and availability of bumble bee‐visited plants (Loffland et al. 2017; Tarbill, White, and Sollmann 2023) in the Sierra Nevada Mountains and other parts of California (Mola et al. 2018). Our study, although limited in scope, provides new insights into how fire impacts bumble bee populations in this region, contributing to a broader understanding of the ecological consequences of fire for pollinators.

## 2 Methods

### 2.1 Research Area and Sites

**FIGURE 1:**
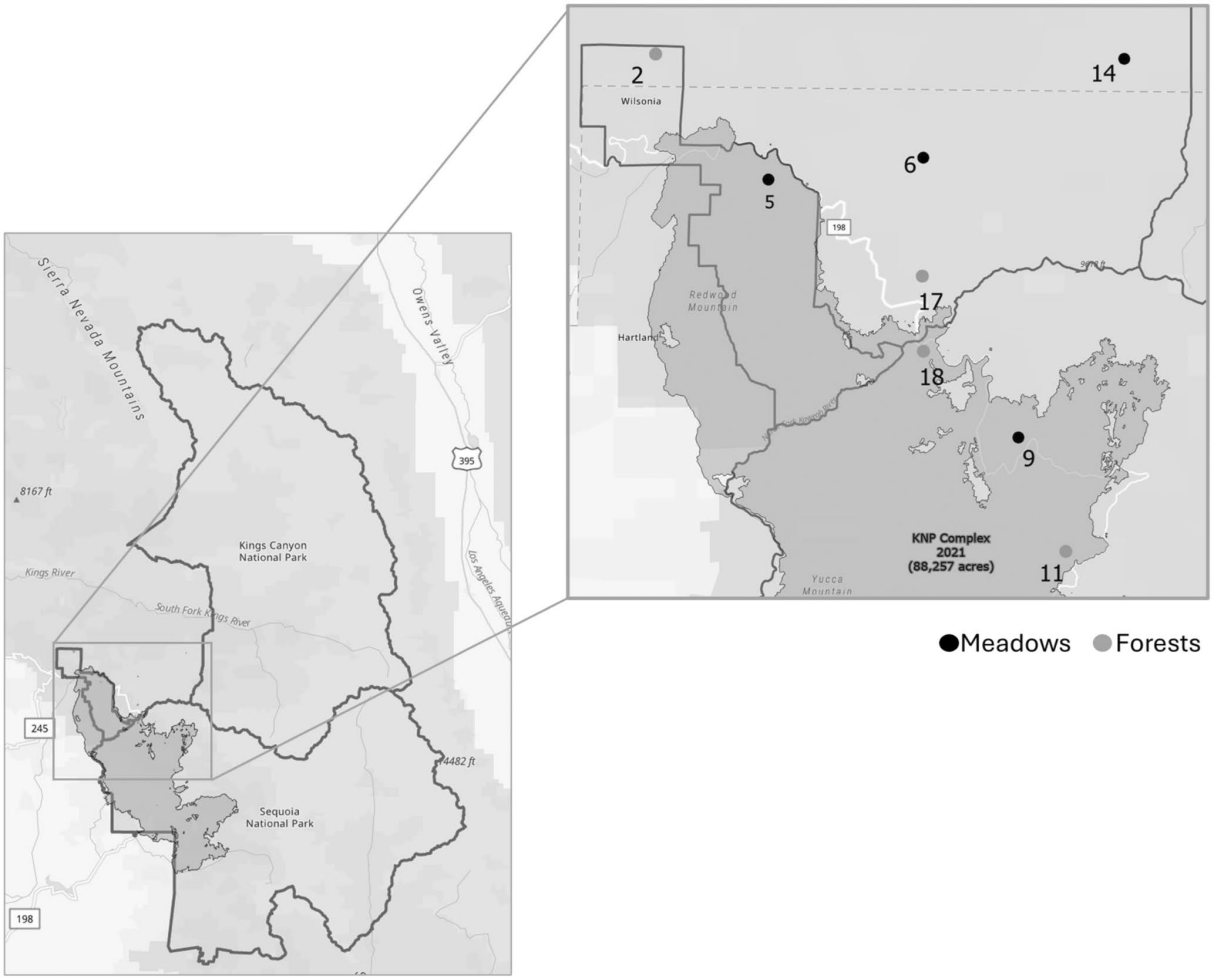
Maps of the study area and sampling sites in the Sequoia and Kings Canyon National Park and the adjacent Sequoia National Forest within the Sierra Nevada Mountains. Burned areas are a part of the KNP Complex Fire, while unburned areas are defined as not having burned in ≥ 10 years. Sampling sites comprised four pairs of burned and unburned areas. These pairs were selected based on similarities in elevation and vegetation habitats using GIS to classify sites as either meadow (> 75% meadow habitat) or forest (> 75% forest habitat) vegetation. Numbers represent site identification (site ID), see Data S2 for more details.

Our study region is located within the Sierra Nevada Mountains in Sequoia and Kings Canyon National Park, and the adjacent Sequoia National Forest (Figure 1). This region has a temperate climate characterized by cool, snowy winters and mild, sunny summers with little to no rainfall. All eight sites were located at 1800–2400 m elevation and are classified as Sierra Nevada US Level III Ecoregion. We worked in areas that burned in 2021 as part of the KNP Complex Fire and also in adjacent unburned areas, with “unburned” defined as not having burned in ≥ 10 years. Data were collected from eight sites comprising four pairs of burned and adjacent unburned sites. All paired sites in our study were environmentally similar in terms of their habitat type and elevation.

We used ArcGIS Pro 3.1.2 (ESRI, Redlands, California, USA) and data layers developed by the University of California Davis Center for Watershed Sciences and USDA Forest Service Pacific Southwest Region to classify sites as either predominantly meadow (> 75% meadow) or forest (> 75% forest) habitat. Each site consisted of a core area with a 500 m radius from a central point, wherein data were collected. To promote the independence of bee data across sites, we maintained a minimum distance of 2 km between the core areas of different sites (see Data S1: Figure S1 for site layout details), as individual bees are unlikely to travel between them (Osborne et al. 2008).

### 2.2 Data Collection

All data collection (bees and floral resources) occurred from 11 to 18 July 2023. At each site, all floral resource data were collected on the same day that bee surveys were performed, and paired sites were both visited on the same day. Data were collected on sunny days without high winds or other adverse weather conditions. Floral resource data were collected along 100 m transects running through the center of each site's core area within a set of 11–15 quadrats (1 m^3^), each separated by a minimum of 10 m. Within each quadrat, we estimated the proportion of area covered by flowering vegetation. We focused on plants that were currently in bloom. Based on visual inspection, we identified these plants at the genus level or, when possible, at the species level. We also validated that all species in our data set occur in the region based on regional field guides and the primary literature. Overall percent floral cover and flowering plant species richness were calculated for each site by averaging and summing the values of all survey quadrats. The percent floral cover in the site was subsequently categorized on a scale from 1 to 6 (1: 0%–5%, 2: 5%–25%, 3: 26%–50%, 4: 51%–75%, 5: 76%–95%, 6: 96%–100%; Mola et al. 2018).

For bumble bee data collection, we first walked haphazardly through each site core between 9:00–16:00 h and collected *B. vosnesenskii* workers. This is overwhelmingly the most common bumble bee species in California, including in the Sierra Nevada Mountains (Thorp, Horning, and Dunning 1983; Loffland et al. 2017; Fisher et al. 2022; Tarbill, White, and Sollmann 2023). *B. vosnesenskii* were collected at sites during 2‐h sampling periods or until 12 individuals were captured. *B. vosnesenskii* were netted, transferred to chilled vials for up to 1 h, photographed using iPhone cameras, and weighed (Fuzion Digital Milligram Scale, 50 g × 0.001 g). Body size data were subsequently extracted from photographs by measuring the lengths of the marginal cells of both wings and then averaging these values. This value is strongly correlated with overall body size in bumble bees and is a commonly used measure of body size in this bee group (Owen 1988). Additionally, we conducted 30‐min point counts to estimate bee abundance at each site. All bee abundance surveys took place between 10:00 and 14:00 h. Here, the floral resource transects described above were re‐walked by an observer who counted every individual bumble bee (all species and any caste) and honey bee ( *Apis mellifera* ; all workers) observed, each over a 30‐min period.

### 2.3 Statistical Analyses

All statistical analyses were performed in R version 4.2.3 (R Core Team 2023), and only *p*‐values < 0.05 were considered significant. All results were visualized with the “ggplot2” package version 3.5.0 (Wickham 2016). We used Spearman's correlation analyses (for non‐parametric data) to examine correlations between body size and body mass. We used generalized linear mixed models (GLMMs) to explore how factors such as burn history (burned vs. unburned), habitat type (forest vs. meadow), floral cover, and honey bee abundance were associated with responses ( *B. vosnesenskii* body size and body mass, as well as bumble bee abundance). GLMMs were performed with the glmer function in the R package lme4 version 1.1–35.1 (Bates et al. 2015). The best‐fit model for our data was selected based on Akaike's Information Criterion (AIC) using the “dredge” command within the MUMIn package version 1.47.5 (Barton 2023). Following model selection, factors of interest were analyzed by performing Likelihood Ratio Tests (LRT) comparing the models with factors to a null model without these factors. Post hoc t‐tests were conducted using Tukey's multiple comparisons of means. Models include elevation as a random variable. A one‐way analysis of variance (ANOVA) was employed to compare data on floral cover according to burned category.

## 3 Results

**FIGURE 2:**
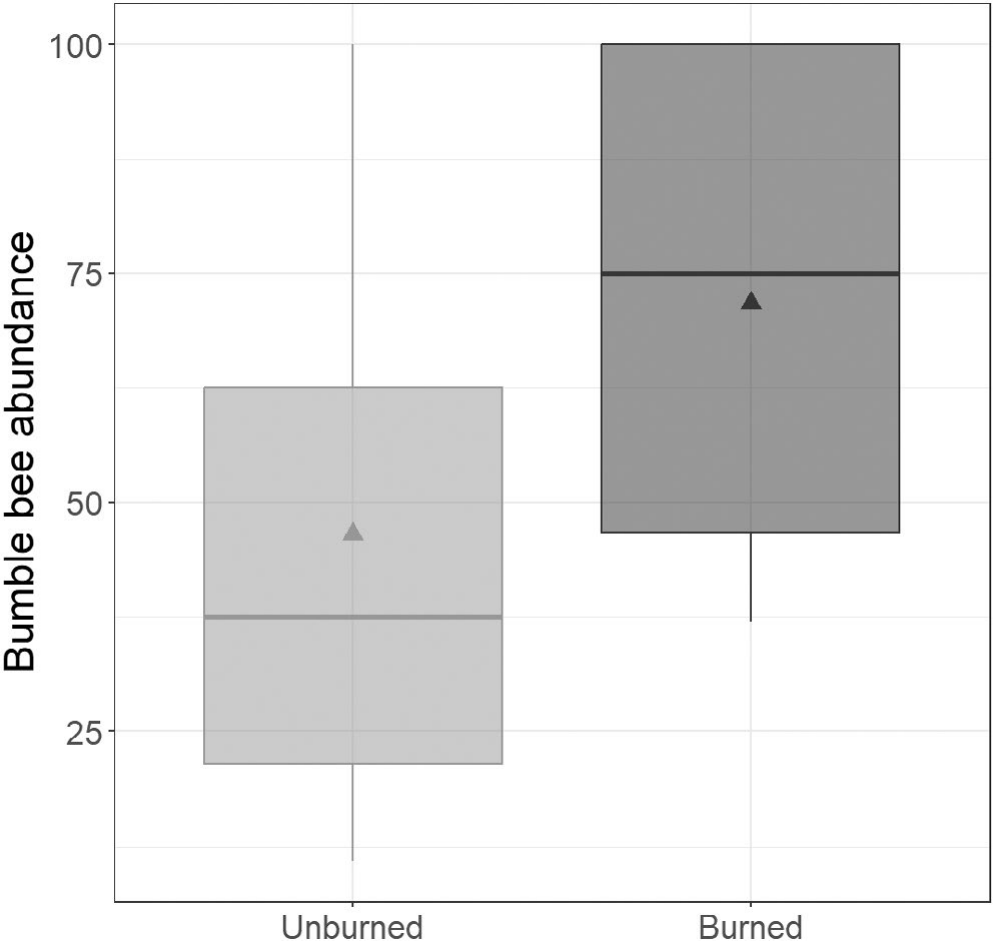
Bumble bee abundance as a function of the burned history. *x*‐axis, burned categories (unburned: Mean ± SEM 46.50 ± 2.87 bees observed; burned: 71.75 ± 1.95 bees observed); *y*‐axis, bees observed. Burned category impacted bumble bee abundance (Tukey's post‐doc: Unburned vs. burned: *q*‐value < 0.0001). Boxplot rectangles show the lower 25% quartile, median (horizontal line), and upper 75% quartile of data, while the lower and upper lines show the 5% and 95% values, respectively, of the data. The triangles represent the means.

**FIGURE 3:**
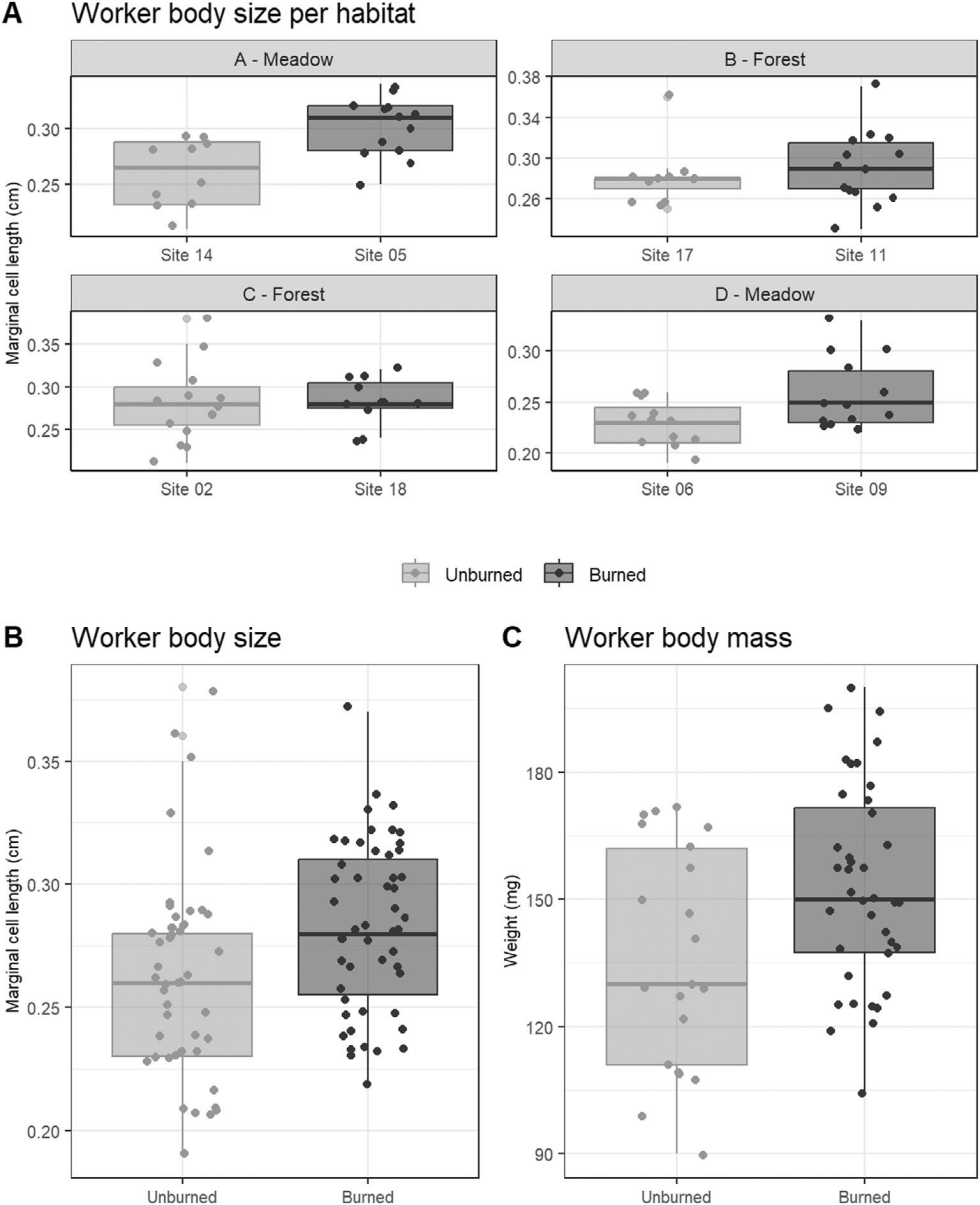
Bumble bee body size and body mass were collected in eight sites comprising four pairs of adjacent burned and unburned areas. These pairs were selected based on similarities in elevation and vegetation habitats. (A) Effects of fire on worker body size in different vegetation habitats. (B) Bumble bee body size as a function of the burned history; *x*‐axis, burned categories (unburned: Mean ± SEM 0.264 ± 0.011 cm; burned: 0.281 ± 0.009 cm); *y*‐axis, length of the second marginal cell of the wing as a proxy for body size. Burned category impacted body size (Tukey's post‐doc: Unburned vs. burned: *q*‐value = 0.034). (C) Effects of fire on worker body mass in different habitats. Body mass as a function of burned category; *x*‐axis, burned categories (unburned: Mean ± SEM 136.523 ± 0.492 mg; burned: 153.179 ± 0.307 mg); *y*‐axis, worker body mass. Burned category impacted body mass (Tukey's post‐doc: Unburned vs. burned: *q*‐value = 0.028).

The model explaining bumble bee abundance indicated a significant effect of burn history (GLMM, χ^2^ = 208.67, *p* < 0.0001; Table 1). Abundance was significantly higher in burned areas (71.75 ± 1.95 SEM) than in unburned areas (49.50 ± 2.87 SEM; Tukey's post hoc test, *q* < 0.0001; Figure 2). *B. vosnesenskii* were also larger and heavier in burned areas compared to unburned areas, regardless of habitat type (Table 1). The body size of *B. vosnesenskii* was significantly affected by burn history (GLMM, χ^2^ = 4.371, *p* = 0.036), with bees in burned areas being larger (0.283 ± 0.009 SEM cm) than those in unburned areas (0.264 ± 0.011 SEM cm; Tukey's post hoc test, *q* = 0.034; Figure 3A,B). Similarly, *B. vosnesenskii* body mass was also significantly influenced by burn history (GLMM, χ^2^ = 3.697, *p* < 0.05), with heavier individuals found in burned areas (153.179 ± 0.307 SEM mg) compared to unburned areas (136.523 ± 0.492 SEM mg; Tukey's post hoc test, *q* = 0.028; Figure 3C). There was a positive correlation between body size and mass (rho = 0.55, *p* < 0.001; Data S1: Figure S2).

**TABLE 1:** Results are from the best‐fitting models. *p*‐values in bold are significant at *p* < 0.05.

Variable response	Analysis	Model	Test statistic	*p*	Direction
Floral percentage cover	Gamma GLMM	Flora relative percentage ~ + random variable “elevation”	na	na	na
Floral richness	Gamma GLMM	Flora richness ~ + random variable “elevation”	na	na	na
Abundance	Gamma GLMM	Bumble bee abundance ~ burned category (Unburned vs. Burned) * site + random variable “elevation”	*z* = −1.844	**< 0.0001**	Unburned < Burned
Body size	Gamma GLMM	Body size ~ burned category (Unburned vs. Burned) + random variable “elevation”	*z* = −2.114	**0.034**	Unburned < Burned
Body mass	Gamma GLMM	Body mass ~ burned category (Unburned vs. Burned) + random variable “elevation”	*z* = −2.199	**0.027**	Unburned < Burned

**FIGURE 4:**
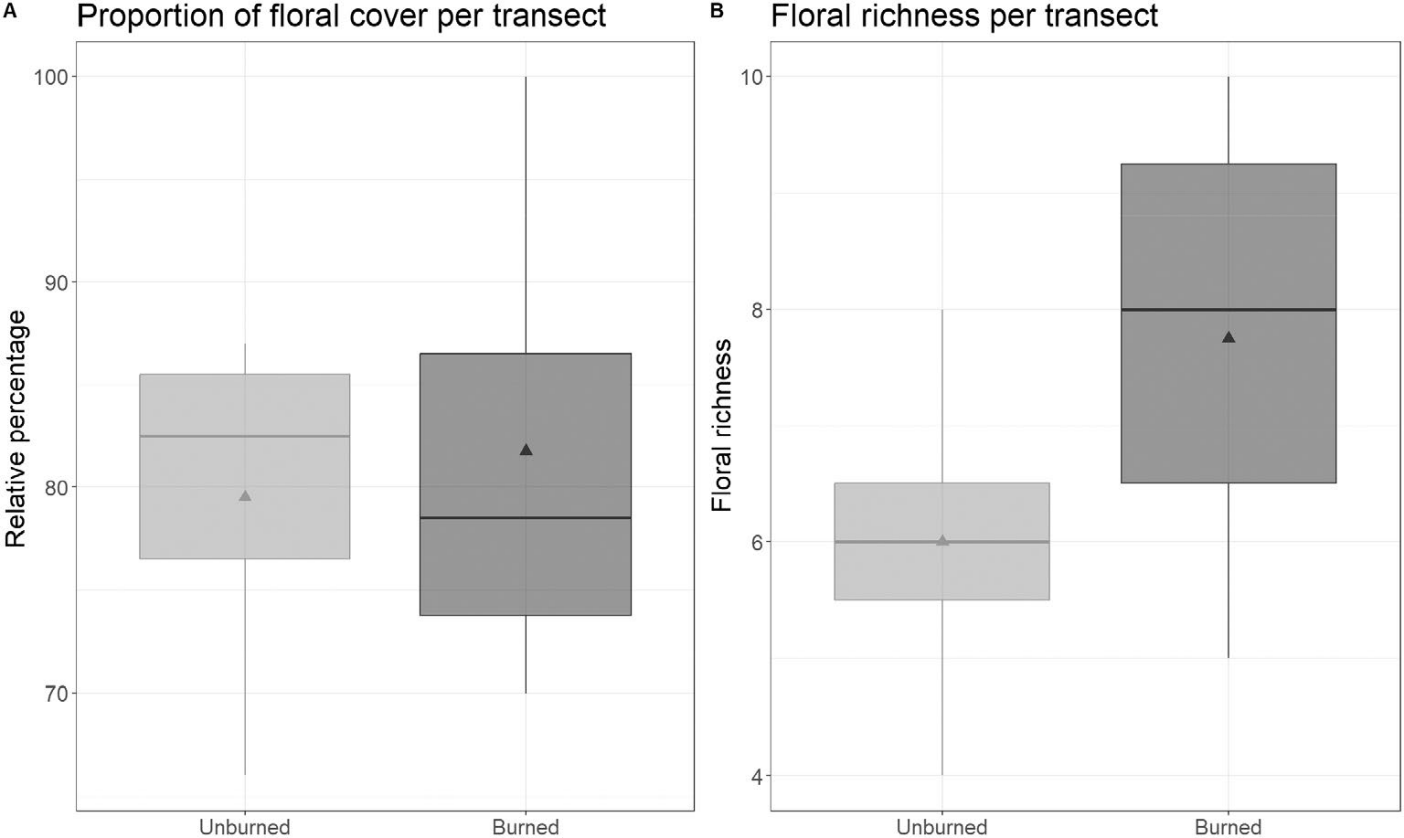
Floral resource data were collected along 100 m transects running through the center of each site core. (A) A floral percent of the ground covers in unburned and burned areas. The relative percentage of floral plants in each site was not impacted by fire (unburned: Mean ± SEM 79.50 ± 0.53 relative percentage; burned: 81.75 ± 0.72 relative percentage). (B) Floral richness in unburned and burned areas. Floral richness in each site was not impacted by fire (unburned: Mean ± SEM 6 ± 0.33 floral richness; burned: 7.75 ± 0.39 floral richness). Boxplot rectangles show the lower 25% quartile, median (horizontal line), and upper 75% quartile of data, while the lower and upper lines show the 5% and 95% values, respectively, of the data. The triangles represent the means.

The only ecological difference between burned and unburned sites was floral species composition. No significant differences were found in floral resource availability (79.50% ± 0.53% SEM in unburned vs. 81.75% ± 0.72% SEM in burned areas; ANOVA, *p* = 0.79; Figure 4A) or floral species richness (6 ± 0.33 SEM in unburned vs. 7.75 ± 0.39 SEM in burned areas; ANOVA, *p* = 0.25; Figure 4B). Honey bee abundance did not differ significantly between burned (1.50 ± 0.71 SEM) and unburned sites (20.75 ± 2.60 SEM), and no association was found between bumble bee and honey bee abundance (Data S1: Figure S3).

## 4 Discussion

As climate change continues to escalate the threat and scale of wildfires (Jones et al. 2022), it will become increasingly important to understand fire's impacts on flora and fauna, including pollinators like bumble bees *Bombus* spp. (Roy and Sparks 2000; Potts et al. 2010; Bartomeus et al. 2011; Goulson et al. 2015). The available evidence indicates that fire can affect bees at the individual, population, and community levels, with some studies even suggesting potential benefits (Ponisio et al. 2016; Carbone et al. 2019; Galbraith et al. 2019). Rapid increases in the intensity of fires beyond what these ecosystems can endure, however, have altered this dynamic, in some cases transforming what were once beneficial events into harmful ones. This intensification is mainly driven by a century of fire suppression (Stephens and Ruth 2005), prolonged drought conditions (Allen et al. 2010), and climate change (Westerling et al. 2006; Abatzoglou and Williams 2016), resulting in fires of unprecedented severity. Consequently, the mechanisms driving these responses remain insufficiently understood. Variables such as magnitude of fire severity (Simanonok and Burkle 2020), the frequency and timescales on which fires occur (Mola et al. 2020), and the surrounding habitat types (Loffland et al. 2017; Johnson et al. 2023) likely play critical roles in shaping bee population dynamics post‐fire. Ongoing studies that examine the broader impacts of fire on pollinators, particularly within fire‐prone ecosystems, are important for gaining a more comprehensive understanding of the consequences of increasing wildfires.

Our study, which focused on how the KNP Complex fire affected the bumble bee *B. vosnesenskii* in the southern Sierra Nevada Mountains, is limited in that we only examined the consequences of one fire complex at one time point within one season, with relatively low sample sizes. Wildfires often have strongly positive impacts on floral diversity and abundance (Lamont and Downes 2011; Richardson and Wagenius 2022; Beck, Waananen, and Wagenius 2023), but we did not observe this pattern in our study, which may be explained by the low number of study sites. We also did not see any mediating influence of habitat type (i.e., whether a pair of burned and unburned sites were predominantly meadow or forest) on the impacts of fire on *B. vosnesenskii* size or bumble or honey bee abundance. Here, we anticipated that fire's impacts in meadow habitat might not be as pronounced as in forested areas, given that meadows already tend to be very high‐quality, flower‐rich habitats for bumble bees in the Sierra Nevada Mountains (Hatfield and LeBuhn 2007; Aldridge et al. 2011; Loffland et al. 2017). Even though we did not find any supporting evidence for this idea, we cannot disentangle whether this was because there is truly no interaction between habitat type and fire or if we simply did not have the power in our study to detect this.

Cautiously, our findings align with previous research suggesting that bumble bees, including *B. vosnesenskii* , may navigate forest habitats more effectively than previously assumed (Mola et al. 2021). Bumble bees will continue to forage in unburned areas, even when nearby burned areas offer richer floral resources (Loffland et al. 2017). This behavior underscores the complexity of bee‐fire interactions and highlights the need for further research to unravel how fire dynamics influence pollinator ecology across various habitat types. We do, however, have greater confidence in our finding that burned areas can contain more, larger‐bodied *B. vosnesenskii* , given that we detected these effects despite our relatively low sample sizes. Areas with higher bumble bee abundances may either be attracting foraging workers from nearby regions or supporting larger local populations, possibly because colonies can gather more food and produce more offspring (Heinrich 2004; Williams, Regetz, and Kremen 2012; Rotheray, Osborne, and Goulson 2017; Hemberger et al. 2020). Even if fire has positive impacts on bumble bees, it is crucial to consider potential trade‐offs. Wildfires might result in colony losses, given that they tend to happen later in the foraging season when colonies are more mature. This might negatively impact local population dynamics, particularly if it limits the production of new reproductives (queens and males). The net benefit of increased foraging resources relative to the risks of colony loss during wildfire events is unclear and requires further study.

In light of our findings, it is important to consider the potential for fire to affect bumble bee populations through changes in floral resources and indirect mechanisms, such as alterations in nesting site availability and changes in microclimatic conditions post‐fire. While we did not find strong evidence of a mediating influence of habitat type or floral resource availability in this study, the possibility remains that fire‐induced shifts in plant community composition (which we did observe) and resource distribution at finer scales were undetected by our sampling methods. Additionally, the impacts of fire on soil chemistry (Hosseini et al. 2017; Liu et al. 2018; but see Agbeshie et al. 2022) and subsequent effects on nectar and pollen composition (Lau and Stephenson 1993; Burkle and Irwin 2010; Hoover et al. 2012; Atasay et al. 2013; Araújo and Rocha‐Filho 2019; Vaudo et al. 2022; Akter and Klečka 2022; reviewed in David, Storkey, and Stevens 2019) could have important implications for bumble bee nutrition and reproductive success. Future research should aim to disentangle these complex interactions by examining multiple fire regimes, habitat types, and time points post‐fire to understand better how fire shapes bumble bee ecology across different landscapes. Although we did not detect an influence of fire on floral resource availability in our study, based on the strong positive relationships between fire, food availability, and bumble bees that have been detected in previous studies (e.g., Lazarina et al. 2016; LoPresti et al. 2018; Mola et al. 2020), we posit that these relationships may also exist in the Southern Sierra Nevada Mountains, and they were not able to be detected in our study based on our sampling design. Alternatively, the increased abundance of bumble bees we observed in burned areas may be driven by the greater availability of nest sites post‐burn. Fires can create more open ground and cavities, ideal for bumble bee nesting (Goulson et al. 2002; Mola et al. 2020). Future studies should aim to explore how changes in nesting opportunities, mediated by fire, might also influence colony survival and reproduction.

Given the relatively small scale of our study, many additional questions remain, such as whether the bees we sampled were produced by colonies located within our study sites or were drawn in from surrounding areas. It is also unclear how the timeframe in which we sampled (2 years after the KNP Complex fire) affected our findings and how the impacts of fire in this area differ based on the number of years since the area burned. Moreover, we do not know the larger consequences of the detected effects of fire on bumble bees, such as whether burned areas support higher bumble bee effective population sizes. As wildfires continue to increase, the significance of the ecological and evolutionary consequences of fire on bumble bee populations, particularly in terms of reproductive success and population health, becomes more relevant for understanding the future of this important pollinator group. Our study, although limited, contributes to the growing body of evidence that fire can have some putatively beneficial impacts on bumble bees.

## 5 Conclusion

Given the significance of *B. vosnesenskii* as a pollinator in the Sierra Nevada Mountains, understanding its response to wildfires is crucial. Our findings suggest that *B. vosnesenskii* may exhibit resilience in the face of fire, potentially benefiting from the altered habitat conditions. The broader ecological and evolutionary implications of this adaptation, however, particularly under the increasing frequency and intensity of wildfires driven by climate change, require more in‐depth study. Our limited study contributes to the growing knowledge on how *B. vosnesenskii* is shaped by fire‐altered landscapes.

## Author Contributions

**Claudinéia P. Costa:** conceptualization (lead), data curation (lead), formal analysis (lead), funding acquisition (supporting), investigation (lead), methodology (lead), project administration (equal), writing – original draft (lead), writing – review and editing (lead). **Natalie Fischer:** data curation (supporting), methodology (equal), writing – review and editing (equal). **Melissa Arellano:** data curation (supporting), writing – review and editing (supporting). **Claudette C. Torres:** data curation (supporting), writing – review and editing (supporting). **S. Hollis Woodard:** funding acquisition (lead), supervision (lead), writing – review and editing (equal).

## Conflicts of Interest

The authors declare no conflicts of interest.

## Supporting information


Data S1.

Data S2.

## Data Availability

All data files and codes can be found in the primary author's GitHub (https://github.com/claudinpcosta/2024‐Wildfires.Bees). Data are also available on Dryad at https://doi.org/10.5061/dryad.1c59zw463.
