# Supplementary material for: Fire Is Associated With Positive Shifts in Bumble Bee ( Bombus vosnesenskii ) Body Size and Bee Abundance in the Southern Sierra Nevada Mountains
Source: Ecol Evol. 2025 Apr 7;15(4):e70821. doi: 10.1002/ece3.70821 (PMC11974454; doi:10.1002/ece3.70821)
Supplement: Supplementary file 1 — Data S1. [file ECE3-15-e70821-s001.docx]

**Supplementary Information for:**

Costa CP, Fischer N, Arellano M, Torres C, Woodard SH. Bumble bees are larger and more abundant in recently-burned areas of the Southern Sierra Nevada Mountains

**ADDITIONAL FILES:**

All data files and codes are provided by GitHub (<https://github.com/claudinpcosta/2024-Wildfires.Bees>).

**MATERIALS AND METHODS**

**(a) Site and Landscape:**

We established replicated sites in burned and unburned areas. A collection site is a 0.5 km (~ 0.78 km2) center point with a 2.5 km radius buffer around it, and then they can overlap. Collectors tried to visit two collection sites daily, including one site in burned and another in non-burned areas.

0.5 km

2 km

**Figure S1** | **Collection area design**. A collection site is a 0.5 km center point with a 2.5 km radius buffer around it.

**RESULTS**

**(a) Floral resource availability at sites.**

**Table S1.** Flowering plant species are present in the unburned and burned sites in Sequoia and Kings Canyon National Park and the adjacent Sequoia National Forest within the Sierra Nevada Mountains. This list of plants contains only species validated based on regional field guides and the primary literature for bumble bee visitation. Cells highlighted in light gray represent species found only in Burned sites. Cells highlighted in dark gray represent species found only in Unburned sites.

|  | **Burned** | | | | **Unburned** | | | |
| --- | --- | --- | --- | --- | --- | --- | --- | --- |
|  | Forest | Forest | Meadow | Meadow | Meadow | Meadow | Forest | Forest |
|  | Site 18 | Site 11 | Site 05 | Site 09 | Site 14 | Site 06 | Site 02 | Site 17 |
| *Bistorta bistortoides* |  |  |  | ✓ |  | ✓ | ✓ |  |
| *Castilleja applegatei* | ✓ |  |  |  |  |  |  |  |
| *Ceanothus cordulatus* |  |  | ✓ | ✓ |  |  |  |  |
| *Clarkia rhomboidea* | ✓ | ✓ |  |  |  |  |  |  |
| *Dodecatheon redolens* |  |  |  | ✓ |  | ✓ | ✓ |  |
| *Draperia systyla* |  | ✓ |  |  |  |  |  |  |
| *Eriogonum wrightii* | ✓ |  |  |  |  |  |  |  |
| *Erysimum capitatum* | ✓ |  |  |  |  |  |  |  |
| *Erythranthe guttatus* |  |  |  | ✓ |  |  |  | ✓ |
| *Hosackia crassifolia* |  |  |  |  |  | ✓ |  |  |
| *Lupinus* |  |  | ✓ | ✓ |  |  | ✓ | ✓ |
| *Mertensia ciliata* |  |  |  | ✓ |  |  | ✓ | ✓ |
| *Monardella odoratissima* |  |  | ✓ |  |  |  |  |  |
| *Phacelia ramosissima* | ✓ | ✓ |  |  | ✓ |  |  | ✓ |
| *Potentilla gracilis* |  |  |  | ✓ | ✓ |  | ✓ | ✓ |
| *Prunus emarginata* |  |  | ✓ |  |  |  |  |  |
| *Ranunculus* |  |  |  |  |  | ✓ |  |  |
| *Solanum xanti* | ✓ |  |  |  | ✓ |  |  |  |
| *Veratrum californicum* |  |  |  |  |  |  | ✓ |  |

**(b) Honey bee abundance.**

**
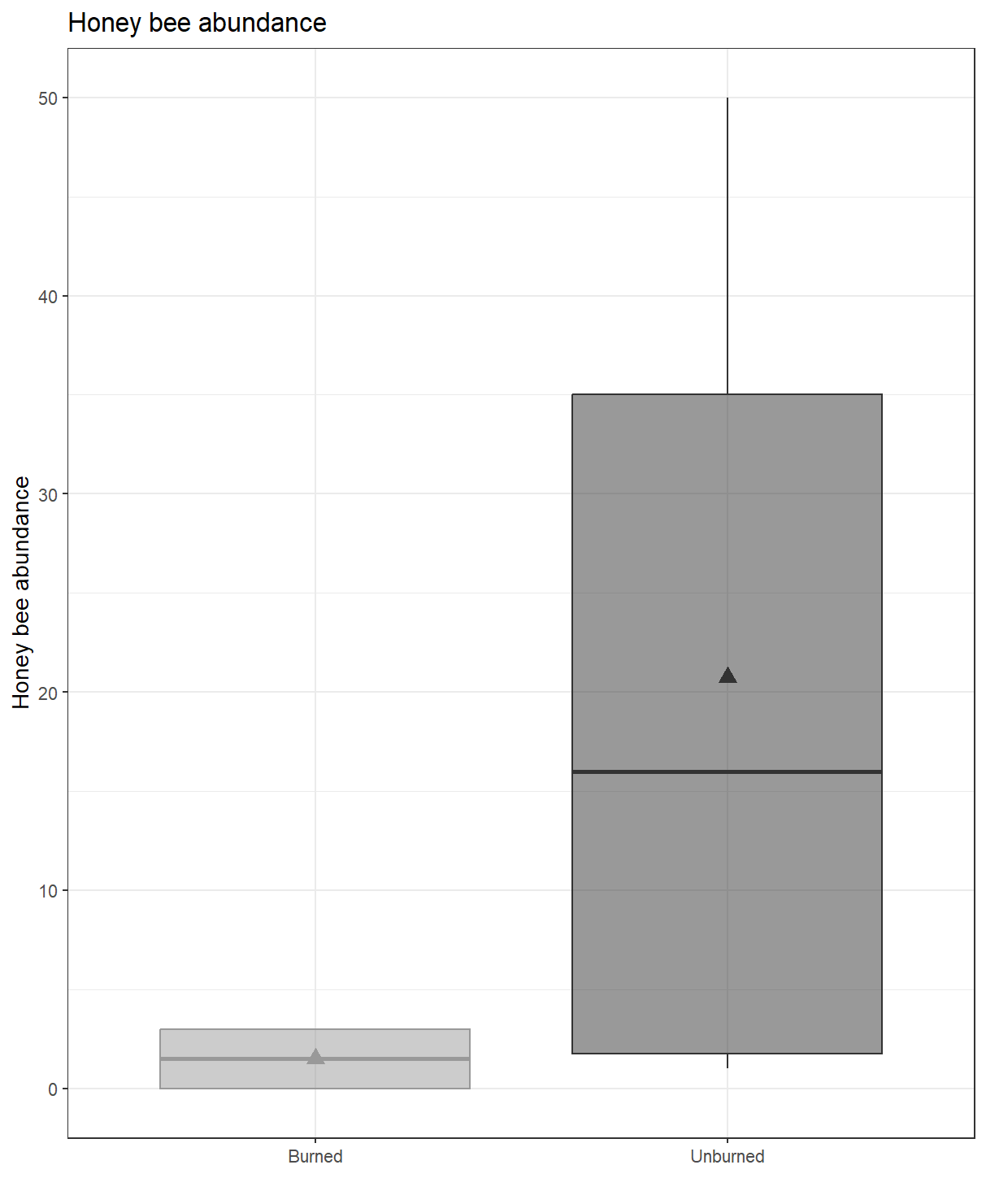

Figure S2** | **Honey bee abundance as a function of the burned history**. x-axis, burned categories (burned: mean ± s.e.m. 1.50 ± 0.71 bees observed; unburned:20.75 ± 2.60 bees observed); y-axis, bees observed. Boxplot rectangles show the lower 25% quartile, median (horizontal line), and upper 75% quartile of data, while the lower and upper lines show the 5 and 95% values, respectively, of the data. The triangles represent the means.

**(c) Correlation between body mass and body size.**

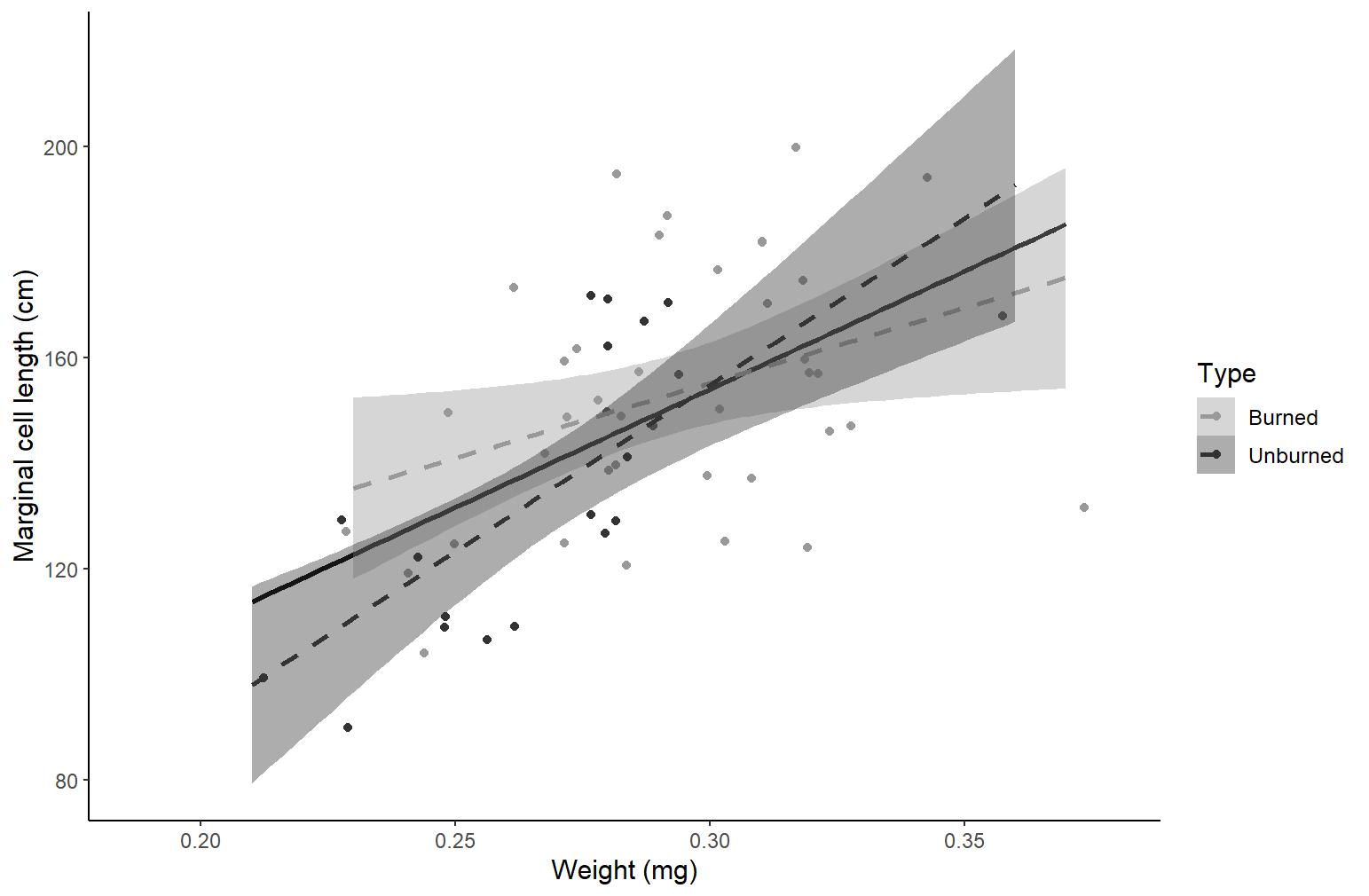


**Figure S3** | **Relationship between body mass and body size** (Spearman: rho = 0.55, p < 0.001). Dashed lines represent regression lines for each group, and the solid line represents the regression line for all combined samples.
